# Supplementary material for: Target Trial Emulation Using Hospital-Based Observational Data: Demonstration and Application in COVID-19
Source: Life (Basel). 2023 Mar 13;13(3):777. doi: 10.3390/life13030777 (PMC10053871; doi:10.3390/life13030777)
Supplement: Supplementary file 1 [file life-13-00777-s001.zip › life-2208626-supplementary.pdf]

Life Journal

Supplementary Material

Martinuka O, von Cube M, Hazard D, Marateb HR, Mansourian M, Sami R, Hajian MR, Wolkewitz M. Target trial emulation using hospital-based observational data: Demonstration and application in COVID-19.

Content

Supplementary Figure S1. Study cohort and emulated target trial flowchart

Supplementary Table S1. STROBE checklist - Statistical Methods for Observational Data on Evaluating Treatment: Demonstration of target trial emulation and application in COVID-19

Supplementary Table S2. Baseline characteristics of patients hospitalized with COVID-19

Supplementary Figure S2. Balance in demographic and clinical characteristics for the emulated cohort based on standardized mean differences in the unweighted and weighted clone samples

Supplementary Table S3. Summary of the corresponding measures of interest taking the parametric approach and estimated weighted results at the end of follow-up (60 days)

Supplementary Figure S3. Effect on hospital length of stay in X-treated (A) and non-X-treated (B) patients

Supplementary Figure S4. Weighted cause-specific cumulative hazards estimated from the Nelson-Aalen estimator for in-hospital death (A) and discharged alive (B) outcomes

Supplementary Figure S5. Weighted cause-specific cumulative incidences estimated using the Aalen-Johansen estimator for in-hospital death (A) and discharged alive (B)

Supplementary Figure S6. Risk differences for in-hospital death and discharged alive outcomes over the follow-up period using the Aalen-Johansen approach

Supplementary Table S4. Summary results of the sensitivity analysis with one-day grace period at the end of follow-up (60 days)

Supplementary Table S5. Summary results of the sensitivity analysis with a grace period (of three days) at the end of follow-up (60 days)

Supplementary Figure S7. Kaplan-Meier estimates of the cumulative probability (%) of in-hospital death

Supplementary Figure S1. Study cohort and emulated target trial flowchart

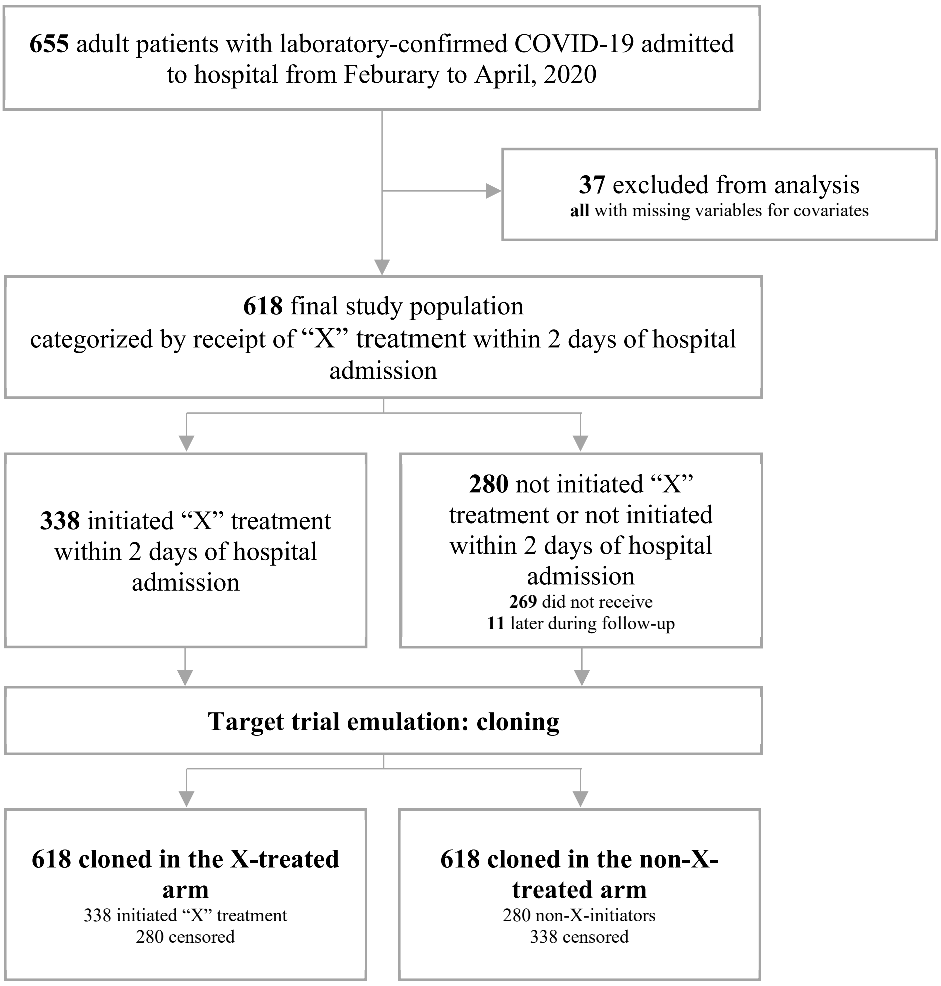

Abbreviation: COVID-19, Coronavirus disease 2019.

Supplementary Table S1. STROBE checklist - Statistical Methods for Observational Data on Evaluating Treatment: Demonstration of target trial emulation and application in COVID-19

	Item No.	Recommendation	Page No.
**Title and abstract**	1	(*a*) Indicate the study's design with a commonly used term in the title or the abstract	1
		(*b*) Provide in the abstract an informative and balanced summary of what was done and what was found	1
**Introduction**			
Background/rationale	2	Explain the scientific background and rationale for the investigation being reported	2
Objectives	3	State specific objectives, including any prespecified hypotheses	2
**Methods**			
Study design	4	Present key elements of study design early in the paper	2
Setting	5	Describe the setting, locations, and relevant dates, including periods of recruitment, exposure, follow-up, and data collection	3-4
Participants	6	Give the eligibility criteria, and the sources and methods of selection of participants. Describe methods of follow-up	3-4
Variables	7	Clearly define all outcomes, exposures, predictors, potential confounders, and effect modifiers. Give diagnostic criteria, if applicable	3,6
Data sources/ measurement	8	For each variable of interest, give sources of data and details of methods of assessment (measurement). Describe comparability of assessment methods if there is more than one group	NA
Bias	9	Describe any efforts to address potential sources of bias	3-7
Study size	10	Explain how the study size was arrived at	NA
Quantitative variables	11	Explain how quantitative variables were handled in the analyses. If applicable, describe which groupings were chosen and why	NA
**Statistical methods**	12	(*a*) Describe all statistical methods, including those used to control for confounding	4-8
		(*b*) Describe any methods used to examine subgroups and interactions	NA
		(*c*) Explain how missing data were addressed	NA
		(*d*) *Cohort study*—If applicable, explain how loss to follow-up was addressed	NA
		(*e*) Describe any sensitivity analyses	8

Continued on next page

**Results**			
Participants	13	(a) Report numbers of individuals at each stage of study—eg numbers potentially eligible, examined for eligibility, confirmed eligible, included in the study, completing follow-up, and analysed	Figure S1
		(b) Give reasons for non-participation at each stage	Figure S1
		(c) Consider use of a flow diagram	Figure S1
Descriptive data	14	(a) Give characteristics of study participants (eg demographic, clinical, social) and information on exposures and potential confounders	Table S2
		(b) Indicate number of participants with missing data for each variable of interest	NA
		(c) Summarise follow-up time (eg, average and total amount)	9
Outcome data	15	Report numbers of outcome events or summary measures over time	8-9
Main results	16	(*a*) Give unadjusted estimates and, if applicable, confounder-adjusted estimates and their precision (eg, 95% confidence interval). Make clear which confounders were adjusted for and why they were included	Table S3
		(*b*) Report category boundaries when continuous variables were categorized	Table S2
		(*c*) If relevant, consider translating estimates of relative risk into absolute risk for a meaningful time period	9, Table S3, Figure 3, S3
Other analyses	17	Report other analyses done—eg analyses of subgroups and interactions, and sensitivity analyses	10, Table S4-S5
**Discussion**			
Key results	18	Summarise key results with reference to study objectives	10-11
Limitations	19	Discuss limitations of the study, taking into account sources of potential bias or imprecision. Discuss both direction and magnitude of any potential bias	11
Interpretation	20	Give a cautious overall interpretation of results considering objectives, limitations, multiplicity of analyses, results from similar studies, and other relevant evidence	10-11
Generalisability	21	Discuss the generalisability (external validity) of the study results	NA
**Other information**			
Funding	22	Give the source of funding and the role of the funders for the present study and, if applicable, for the original study on which the present article is based	12

Supplementary Table S2. Baseline characteristics of patients hospitalized with COVID-19

Characteristic	n
**Overall sample**	618
**Gender**	
Male	384 (62.1 %)
Female	234 (37.9 %)
**Age, years**	
Mean (SD)	57.2
Median [Min, Max]	58.0 [20.0, 92.0]
**O2 saturation [%]**	
Mean (SD)	89 (7.2)
Median [Min, Max]	91 [35, 100]
**Respiratory rate [breaths per minute]**	
Mean (SD)	21.9 (5.2)
Median [Min, Max]	20 [12, 45]
**Creatinine [mg/dL]**	
Mean (SD)	1.20 (1.02)
Median [Min, Max]	0.96 [0.41, 9.89]

Abbreviation: SD, standard deviation.

Supplementary Figure S2. Balance in demographic and clinical characteristics for the emulated cohort based on standardized mean differences in the unweighted and weighted clone samples

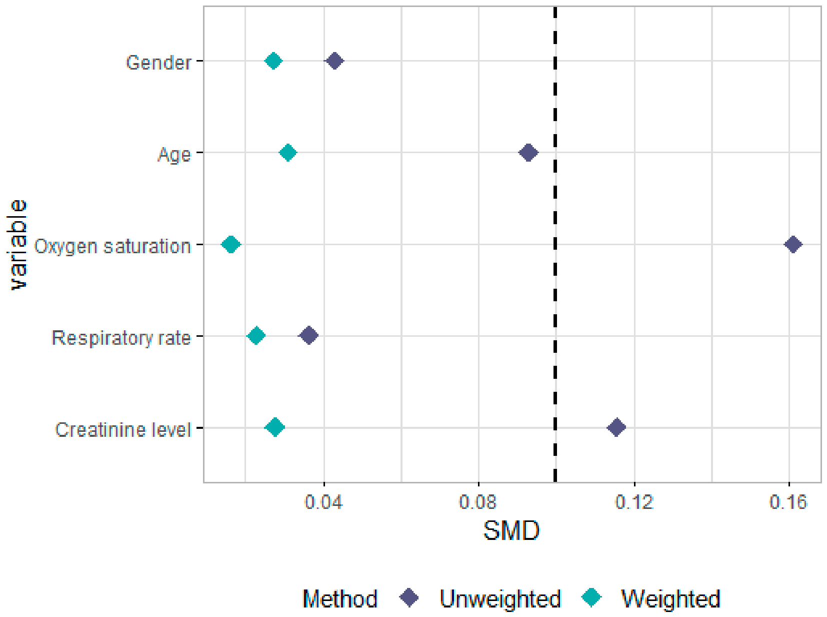

Notes: SMD < 0.1: adequate balance, 0.1-0.25: irrelevant imbalance, >0.25 serious imbalance[1].
Abbreviation: SMD, standardized mean difference.

The standardized differences were examined to ensure that the weights eliminated any imbalance between treatment arms. [1] A threshold below 10% of the standardized mean differences is considered a meaningful balance. [2]

References

1. Willems, S.; Schat, A.; van Noorden, M.S.; Fiocco, M. Correcting for dependent censoring in routine outcome monitoring data by applying the inverse probability censoring weighted estimator. Stat. Methods Med. Res. 2018, 27, 323–335, doi:10.1177/0962280216628900.
2. Stuart, E.A.; Lee, B.K.; Leacy, F.P. Prognostic score-based balance measures can be a useful diagnostic for propensity score methods in comparative effectiveness research. J. Clin. Epidemiol. 2013, 66, S84-S90.e1, doi:10.1016/j.jclinepi.2013.01.013.

Supplementary Table S3. Summary of the corresponding measures of interest taking the parametric approach and estimated weighted results at the end of follow-up (60 days)

Corresponding measure	Mathematical formulation	Results [95% CI]
**Constant hazards**		
Death hazard w/o treatment	λ_{02}	0.010
Discharge hazard w/o treatment	λ_{03}	0.120
Hazard w/o treatment	$\lambda_0 = \lambda_{02} + \lambda_{03}$	0.130
Death hazard with treatment	λ_{12}	0.008
Discharge hazard with treatment	λ_{13}	0.124
Hazard with treatment	$\lambda_1 = \lambda_{12} + \lambda_{13}$	0.131
**Mortality**		
Mortality risk w/o treatment at day 60	$MR_0 = \frac{\lambda_{02}}{\lambda_{02} + \lambda_{03}}$	0.074 [0.031-0.085]
Mortality risk with treatment at day 60	$MR_1 = \frac{\lambda_{12}}{\lambda_{12} + \lambda_{13}}$	0.058 [0.037-0.076]
Mortality risk ratio at day 60	$\frac{\lambda_{12}/\lambda_{02}}{(\lambda_{13} + \lambda_{12})/(\lambda_{02} + \lambda_{03})}$	0.785 [0.535-1.316]
Difference in mortality at day 60	$MR_1 - MR_0$	-0.016 [-0.033, 0.030]
**Occurrence of outcome and treatment effect estimands**		
Hazard ratio of death (treatment *vs.* w/o treatment)	$HR_2 = \frac{\lambda_{12}}{\lambda_{02}}$	0.793 [0.480-1.247]
Hazard ratio of discharge (treatment *vs.* w/o treatment)	$HR_3 = \frac{\lambda_{13}}{\lambda_{03}}$	1.027 [0.806-1.045]
Differences in hazard ratios of death (treatment *vs.* w/o treatment) at day 60	$\lambda_{12} - \lambda_{02}$	-0.002 [-0.005, 0.003]
Differences in hazard ratios of discharge (treatment *vs.* w/o treatment) at day 60	$\lambda_{13} - \lambda_{03}$	0.003 [-0.023, 0.005]
Cumulative risk of death w/o treatment at time t	$CIF_{02} = \frac{\lambda_{02}}{\lambda_0} \times (1 - \exp(-\lambda_0 t))$	0.074 [0.031-0.085]
Cumulative risk of discharge w/o treatment at time t	$CIF_{03} = \frac{\lambda_{03}}{\lambda_0} \times (1 - \exp(-\lambda_0 t))$	0.924 [0.913-0.968]
Cumulative risk of death with treatment at time t	$CIF_{12} = \frac{\lambda_{12}}{\lambda_1} \times (1 - \exp(-\lambda_1 t))$	0.058 [0.037-0.076]
Cumulative risk of discharge with treatment at time t	$CIF_{12} = \frac{\lambda_{13}}{\lambda_1} \times (1 - \exp(-\lambda_1 t))$	0.940 [0.921-0.961]
Risk difference functions for death at day 60	$RD_2 = CIF_{12} - CIF_{02}$	-0.016 [-0.033, 0.030]
Risk difference functions for discharge at day 60	$RD_3 = CIF_{13} - CIF_{03}$	0.016 [-0.031, 0.032]
Risk ratios for death at day 60	$RR_2 = \frac{CIF_{12}}{CIF_{02}}$	0.785 [0.053, 1.309]

Continued on next page

**Corresponding measure**	**Mathematical formulation**	**Results**
Risk ratios for discharge at day 60	$RR_3 = \frac{CIF_{13}}{CIF_{03}}$	1.017 [0.965, 1.035]
**Length of stay**		
Length of stay w/o treatment	$LOS_0 = \frac{1}{\lambda_{02} + \lambda_{03}}$	7.69 days [7.00, 8.39]
Length of stay with treatment	$LOS_1 = \frac{1}{\lambda_{12} + \lambda_{13}}$	7.61 days [7.21, 8.03]
Difference in length of stay	$LOS_1 - LOS_0$	-0.08

Notes: 0 = from non-X-treated state, 1 = from X-treated state.
Abbreviations: CIF, cumulative incidence function; HR, hazard ratio; LOS, length of stay; MR, mortality risk, RD, risk difference; RR, risk ratio; w/o, without.

Supplementary Figure S3. Effect on hospital length of stay in X-treated (A) and non-X-treated (B) patients

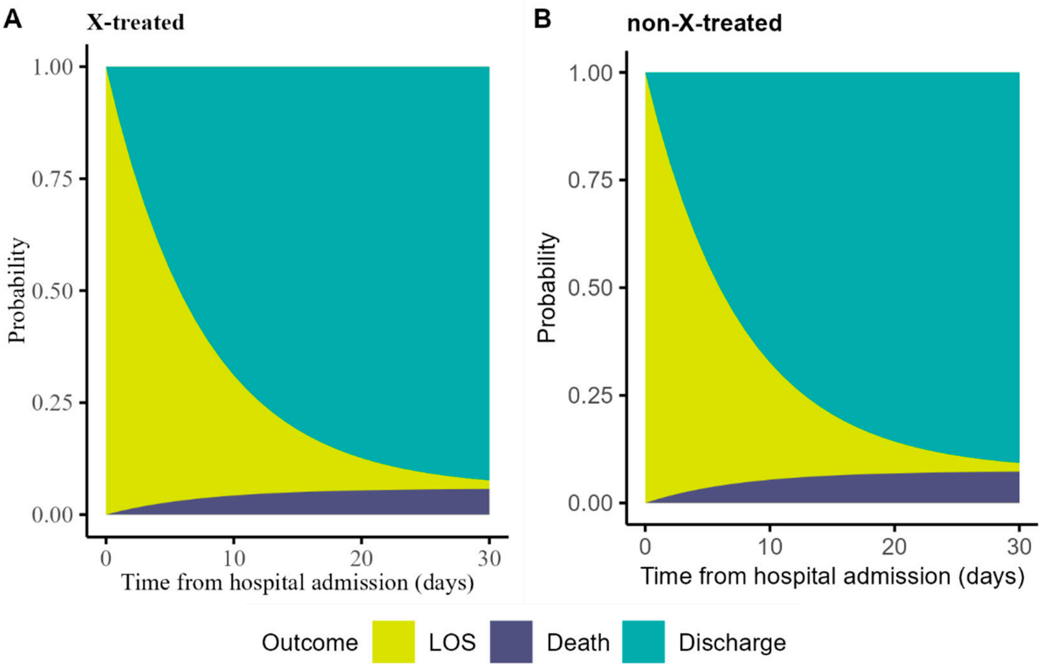

Abbreviation: LOS, length of stay.

Results from the additional analyses

Supplementary Figure S4. Weighted cause-specific cumulative hazards estimated from the Nelson-Aalen estimator for in-hospital death (A) and discharged alive (B) outcomes

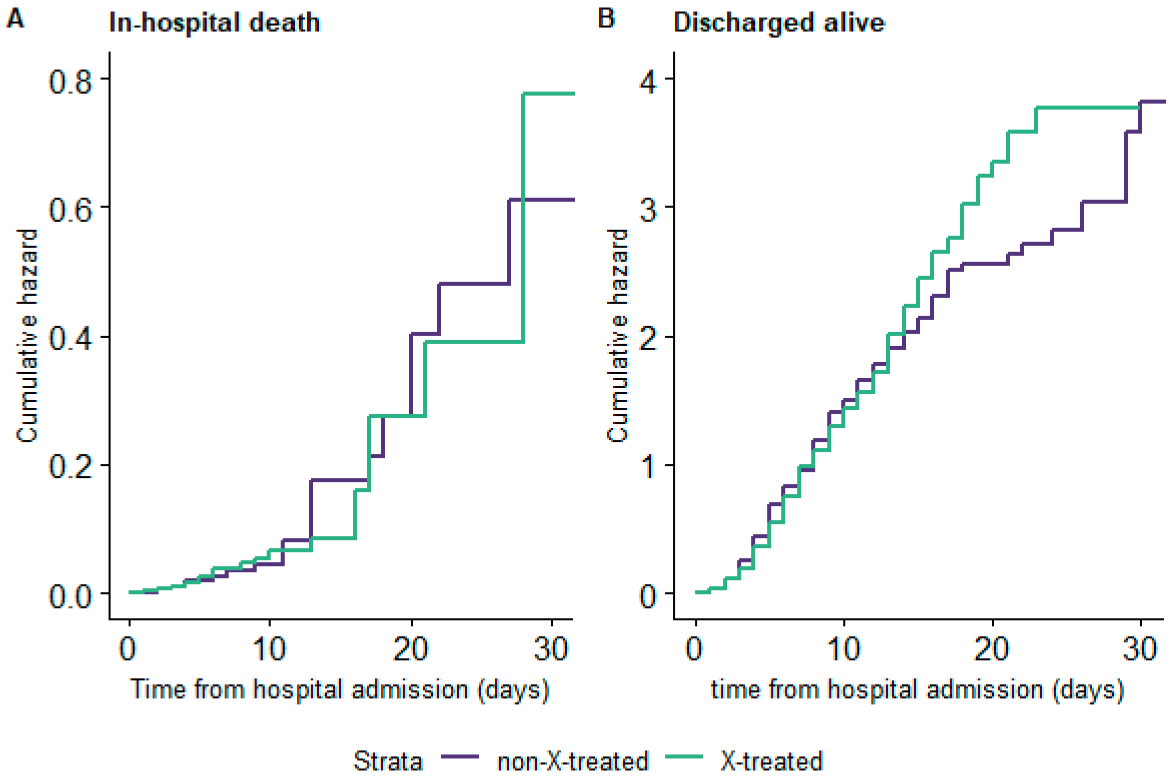

Supplementary Figure S5. Weighted cause-specific cumulative incidences estimated using the Aalen-Johansen estimator for in-hospital death (A) and discharged alive (B)

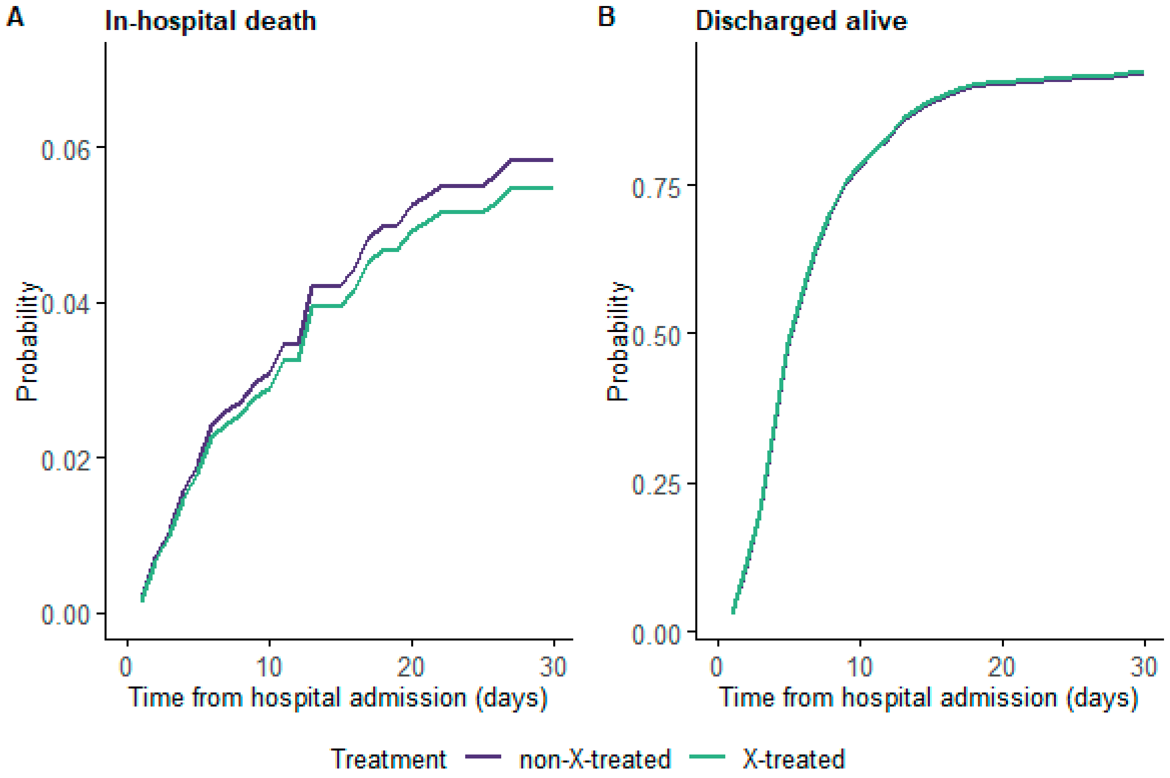

Supplementary Figure S6. Risk differences for in-hospital death and discharged alive outcomes over the follow-up period using the Aalen-Johansen approach

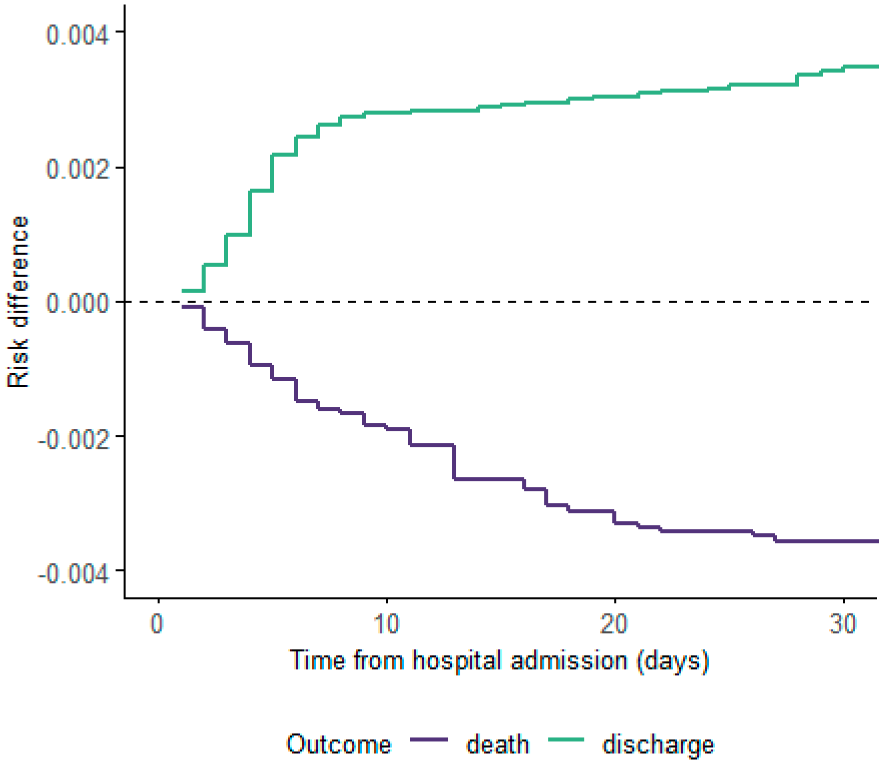

Supplementary Table S4. Summary results of the sensitivity analysis with one-day grace period at the end of follow-up (60 days)

Corresponding measure	Mathematical formulation	Results [95% CI]
**Constant hazards**		
Death hazard w/o treatment	λ_{02}	0.010
Discharge hazard w/o treatment	λ_{03}	0.121
Hazard w/o treatment	$\lambda_0 = \lambda_{02} + \lambda_{03}$	0.130
Death hazard with treatment	λ_{12}	0.007
Discharge hazard with treatment	λ_{13}	0.124
Hazard with treatment	$\lambda_1 = \lambda_{12} + \lambda_{13}$	0.131
**Mortality**		
Mortality risk w/o treatment at day 60	$MR_0 = \frac{\lambda_{02}}{\lambda_{02} + \lambda_{03}}$	0.074 [0.031-0.085]
Mortality risk with treatment at day 60	$MR_1 = \frac{\lambda_{12}}{\lambda_{12} + \lambda_{13}}$	0.055 [0.029-0.068]
Mortality risk ratio at day 60	$\frac{\lambda_{12}/\lambda_{02}}{(\lambda_{13} + \lambda_{12})/(\lambda_{02} + \lambda_{03})}$	0.750 [0.411-1.197]
Difference in mortality at day 60	$MR_1 - MR_0$	-0.018 [-0.041-0.023]
**Occurrence of outcome and treatment effect estimands**		
Hazard ratio of death (treatment *vs.* w/o treatment)	$HR_2 = \frac{\lambda_{12}}{\lambda_{02}}$	0.754 [0.352-1.125]
Hazard ratio of discharge (treatment *vs.* w/o treatment)	$HR_3 = \frac{\lambda_{13}}{\lambda_{03}}$	1.025 [0.801-1.042]
Differences in hazard ratios of death (treatment *vs.* w/o treatment) at day 60	$\lambda_{12} - \lambda_{02}$	-0.002 [-0.006, 0.002]
Differences in hazard ratios of discharge (treatment *vs.* w/o treatment) at day 60	$\lambda_{13} - \lambda_{03}$	0.003 [-0.024, 0.005]
Cumulative risk of death w/o treatment at time t	$CIF_{02} = \frac{\lambda_{02}}{\lambda_0} \times (1 - \exp(-\lambda_0 t))$	0.073 [0.031-0.085]
Cumulative risk of discharge w/o treatment at time t	$CIF_{03} = \frac{\lambda_{03}}{\lambda_0} \times (1 - \exp(-\lambda_0 t))$	0.925 [0.913-0.968]
Cumulative risk of death with treatment at time t	$CIF_{12} = \frac{\lambda_{12}}{\lambda_1} \times (1 - \exp(-\lambda_1 t))$	0.055 [0.029-0.068]
Cumulative risk of discharge with treatment at time t	$CIF_{12} = \frac{\lambda_{13}}{\lambda_1} \times (1 - \exp(-\lambda_1 t))$	0.943 [0.929-0.968]
Risk difference functions for death at day 60	$RD_2 = CIF_{12} - CIF_{02}$	-0.018 [-0.041, 0.023]
Risk difference functions for discharge at day 60	$RD_3 = CIF_{13} - CIF_{03}$	0.018 [-0.024, 0.040]
Risk ratios for death at day 60	$RR_2 = \frac{CIF_{12}}{CIF_{02}}$	0.750 [0.041, 1.196]

Continued on next page

Corresponding measure	Mathematical formulation	Results
Risk ratios for discharge at day 60	$RR_3 = \frac{CIF_{13}}{CIF_{03}}$	1.020<br>[0.973, 1.044]
**Length of stay**		
Length of stay w/o treatment	$LOS_0 = \frac{1}{\lambda_{02} + \lambda_{03}}$	7.66 days<br>[6.89, 8.31]
Length of stay with treatment	$LOS_1 = \frac{1}{\lambda_{12} + \lambda_{13}}$	7.63 days<br>[7.84, 8.71]
Difference in length of stay	$LOS_1 - LOS_0$	-0.03

Notes: 0 = from non-X-treated state, 1 = from X-treated state.
Abbreviations: CIF, cumulative incidence function; HR, hazard ratio; LOS, length of stay; MR, mortality risk, RD, risk difference; RR, risk ratio; w/o, without.

Mean of the weights 1.608 for the treated and 1.837 for the control group.

Supplementary Table S5. Summary results of the sensitivity analysis with a grace period (of three days) at the end of follow-up (60 days)

Corresponding measure	Mathematical formulation	Results [95% CI]
**Constant hazards**		
Death hazard w/o treatment	λ_{02}	0.010
Discharge hazard w/o treatment	λ_{03}	0.121
Hazard w/o treatment	$\lambda_0 = \lambda_{02} + \lambda_{03}$	0.131
Death hazard with treatment	λ_{12}	0.007
Discharge hazard with treatment	λ_{13}	0.124
Hazard with treatment	$\lambda_1 = \lambda_{12} + \lambda_{13}$	0.132
**Mortality**		
Mortality risk w/o treatment at day 60	$MR_0 = \frac{\lambda_{02}}{\lambda_{02} + \lambda_{03}}$	0.076 [0.033-0.088]
Mortality risk with treatment at day 60	$MR_1 = \frac{\lambda_{12}}{\lambda_{12} + \lambda_{13}}$	0.055 [0.034-0.070]
Mortality risk ratio at day 60	$\frac{\lambda_{12}/\lambda_{02}}{(\lambda_{13} + \lambda_{12})/(\lambda_{02} + \lambda_{03})}$	0.728 [0.469-1.176]
Difference in mortality at day 60	$MR_1 - MR_0$	-0.021 [-0.034, 0.022]
**Occurrence of outcome and treatment effect estimands**		
Hazard ratio of death (treatment *vs.* w/o treatment)	$HR_2 = \frac{\lambda_{12}}{\lambda_{02}}$	0.730 [0.415-1.102]
Hazard ratio of discharge (treatment *vs.* w/o treatment)	$HR_3 = \frac{\lambda_{13}}{\lambda_{03}}$	1.026 [0.806-1.040]
Differences in hazard ratios of death (treatment *vs.* w/o treatment) at day 60	$\lambda_{12} - \lambda_{02}$	-0.003 [-0.006, 0.002]
Differences in hazard ratios of discharge (treatment *vs.* w/o treatment) at day 60	$\lambda_{13} - \lambda_{03}$	0.003 [-0.023, 0.005]
Cumulative risk of death w/o treatment at time t	$CIF_{02} = \frac{\lambda_{02}}{\lambda_0} \times (1 - \exp(-\lambda_0 t))$	0.076 [0.033-0.088]
Cumulative risk of discharge w/o treatment at time t	$CIF_{03} = \frac{\lambda_{03}}{\lambda_0} \times (1 - \exp(-\lambda_0 t))$	0.923 [0.910-0.966]
Cumulative risk of death with treatment at time t	$CIF_{12} = \frac{\lambda_{12}}{\lambda_1} \times (1 - \exp(-\lambda_1 t))$	0.055 [0.034-0.070]
Cumulative risk of discharge with treatment at time t	$CIF_{12} = \frac{\lambda_{13}}{\lambda_1} \times (1 - \exp(-\lambda_1 t))$	0.943 [0.927-0.964]
Risk difference functions for death at day 60	$RD_2 = CIF_{12} - CIF_{02}$	-0.021 [-0.039, 0.022]
Risk difference functions for discharge at day 60	$RD_3 = CIF_{13} - CIF_{03}$	0.021 [-0.024, 0.038]
Risk ratios for death at day 60	$RR_2 = \frac{CIF_{12}}{CIF_{02}}$	0.728 [0.047, 1.175]

Continued on next page

Corresponding measure	Mathematical formulation	Results
Risk ratios for discharge at day 60	$RR_3 = \frac{CIF_{13}}{CIF_{03}}$	1.022<br>[0.973, 1.042]
**Length of stay**		
Length of stay w/o treatment	$LOS_0 = \frac{1}{\lambda_{02} + \lambda_{03}}$	7.62 days<br>[6.87, 8.30]
Length of stay with treatment	$LOS_1 = \frac{1}{\lambda_{12} + \lambda_{13}}$	7.60 days<br>[7.84, 8.66]
Difference in length of stay	$LOS_1 - LOS_0$	-0.02

Notes: 0 = from non-X-treated state, 1 = from X-treated state.
Abbreviations: CIF, cumulative incidence function; HR, hazard ratio; LOS, length of stay; MR, mortality risk, RD, risk difference; RR, risk ratio; w/o, without.

Mean of the weights 1.303 for the treated and 1.863 for the control group.

Supplementary Figure S7. Kaplan-Meier estimates of the cumulative probability (%) of in-hospital death

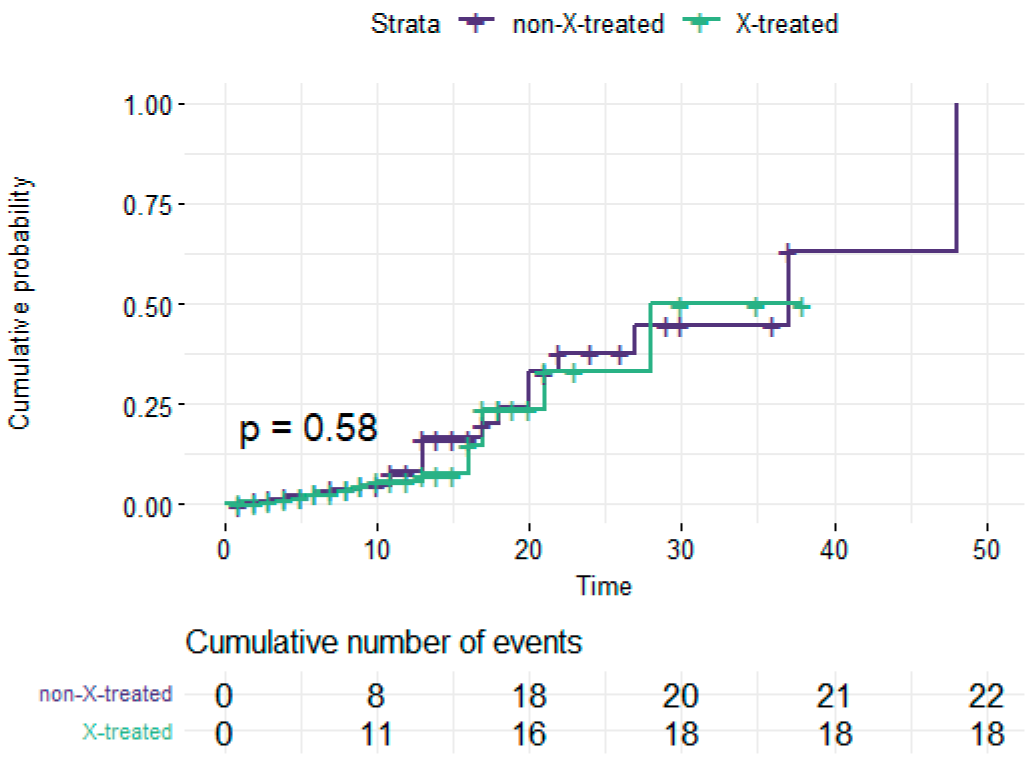

*Using an immortal-time dataset, all treated patients were classified as treated from time zero

The Kaplan-Meier analysis

We used the standard Kaplan-Meier survival analysis (that is, one minus survival function) to calculate the cumulative probability of in-hospital death over time. Competing risk bias was artificially introduced using the Kaplan-Meier method that treats all discharged events as right-censored.
